# Молекулярная генетика в детской эндокринологии: 35 лет исследований

**DOI:** 10.14341/probl13593

**Published:** 2025-05-20

**Authors:** В. А. Петеркова, О. Б. Безлепкина, М. С. Панкратова, И. С. Чугунов, Д. Н. Лаптев, Е. В. Нагаева, Т. Ю. Ширяева, А. А. Колодкина, Л. С. Созаева, Е. В. Титович, А. В. Болмасова, Т. Л. Кураева

**Affiliations:** Национальный медицинский исследовательский центр эндокринологии; Национальный медицинский исследовательский центр эндокринологии; Национальный медицинский исследовательский центр эндокринологии; Национальный медицинский исследовательский центр эндокринологии; Национальный медицинский исследовательский центр эндокринологии; Национальный медицинский исследовательский центр эндокринологии; Национальный медицинский исследовательский центр эндокринологии; Национальный медицинский исследовательский центр эндокринологии; Национальный медицинский исследовательский центр эндокринологии; Национальный медицинский исследовательский центр эндокринологии; Национальный медицинский исследовательский центр эндокринологии; Национальный медицинский исследовательский центр эндокринологии

**Keywords:** детская эндокринология, генетика, Институт детской эндокринологии

## Abstract

Современная детская эндокринология – это рассвет диагностики и лечения на основе научных изысканий в области молекулярной генетики и разработки технических средств диагностики и лечения. В детской клинике Эндокринологического научного центра  РАМН (далее ЭНЦ), позднее -  Институте детской эндокринологии ФГБУ «НМИЦ эндокринологии» Минздрава России молекулярно-генетические исследования, начатые в 1990 году, проводились совместно с Медико-генетическим научным центром им. Н.П. Бочкова,   Институтом иммунологии, а также в кооперации с зарубежными клиниками. С 2001 года в ЭНЦ функционирует лаборатория молекулярной генетики.

## Abstract (en)

Modern pediatric endocrinology represents the dawn of a new era in diagnosis and treatment, based on scientific research in molecular genetics and the development of advanced diagnostic and therapeutic tools. At the Pediatric Clinic of the En docrinology Research Centre (ERC), later the Institute of Pediatric Endocrinology, molecular genetic research began in 1990 in collaboration with the N.P. Bochkov Research Centre for Medical Genetics and the Institute of Immunology. The ERC’s Laboratory of Molecular Genetics has been operational since 2001, conducting research in partnership with foreign clinics and institutions.

Современная детская эндокринология — это рассвет диагностики и лечения на основе научных изысканий в области молекулярной генетики и разработки технических средств диагностики и лечения. В детской клинике Эндокринологического научного центра РАМН (далее ЭНЦ), позднее — Институте детской эндокринологии ФГБУ «НМИЦ эндокринологии» Минздрава России молекулярно-генетические исследования, начатые в 1990 г., проводились совместно с Медико-генетическим научным центром им. Н.П. Бочкова, Институтом иммунологии, а также в кооперации с зарубежными клиниками. С 2001 г. в ЭНЦе функционирует лаборатория молекулярной генетики. Эволюция развития молекулярной диагностики в ЭНЦе представлена на рисунке 1.

**Figure:**
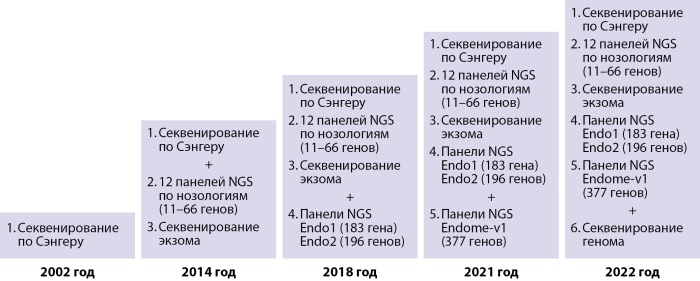
Рисунок 1. Эволюция развития молекулярной диагностики в ЭНЦ.

С началом в 2014 г. благотворительной программы «Альфа-Эндо» появилась возможность проводить молекулярно-генетические исследования методом массового параллельного секвенирования нового поколения. Значительная доля врожденных заболеваний эндокринной системы у детей имеет общие клинические черты, что требует одновременного исследования большого количества генов. В рамках программы были созданы панели, включающие гены, патология которых приводит к развитию определенной группы заболеваний (надпочечниковая недостаточность, гипогонадотропный гипогонадизм и другие). Параллельно продолжилась работа по исследованию генов методом секвенирования по Сэнгеру, исследование частых мутаций.

С 2018 г. в рамках программы «Альфа-Эндо» стало доступно полноэкзомное секвенирование, которое позволило исследовать самые редкие причины эндокринопатий. С целью ускорения получения результатов и удешевления секвенирования была разработана генетическая панель «Эндом», содержащая 377 генов, ответственных за развитие большинства врожденных заболеваний эндокринной системы.

С первых дней существования программы «Альфа-эндо» перед нами стояли две основные задачи: первая — обучение детских эндокринологов применению методов молекулярно-генетической диагностики в ежедневной клинической практике и вторая — доступность этих методов в России для всех детей независимо от места проживания. Эти две задачи успешно осуществлены. В настоящее время образцы крови для исследования доставляются в ЭНЦ курьерской службой после предварительной консультации медицинских заключений специалистами Института детской эндокринологии.

Всего за прошедшие 10 лет в рамках программы «Альфа-Эндо» было проведено исследование свыше 17,5 тысячи образцов биоматериала, количество исследований в разные годы приведено на рисунке 2, а в таблице 1 представлена информация о количестве образцов и использованных методах диагностики.

**Figure:**
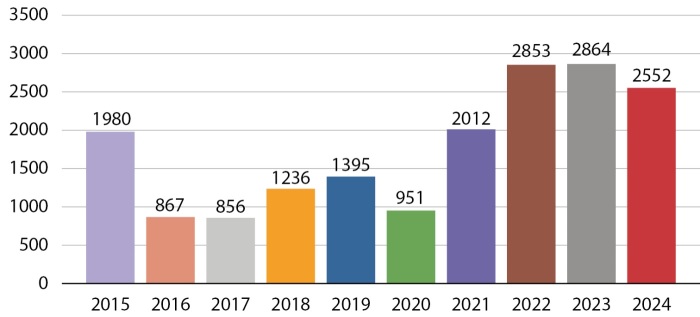
Рисунок 2. Количество молекулярно-генетических исследований в 2015–2024 годы.

**Table:** Таблица 1. Количество исследованных образцов крови по программе «Альфа-Эндо»

Название исследования	Количество образцов
Секвенирование клинического экзома	1515
Секвенирование панели «Эндом»	228
Секвенирование отдельных генов, детекция известных мутаций	3886
Исследование гена CYP21A2 (детекция частых мутаций, полное секвенирование гена)	2470
Другие исследования	2167
Секвенирование панелей генов
Гипопитуитаризм	744
Сахарный диабет — гиперинсулинизм	3179
Примордиальный нанизм	75
Аденомы гипофиза	661
Врожденный гипотиреоз	468
Гиперпаратиреоз	234
Гипогонадотропный гипогонадизм	457
Надпочечниковая недостаточность	291
Нарушения формирования пола	749
Рахитоподобные заболевания	363
Несовершенный остеогенез	54
Наследственные липодистрофии	25

В настоящее время используются уникальные панели известных генов, нарушения в которых вызывают определенные группы эндокринных заболеваний:

Сахарный диабет — гиперинсулинизм: ABCC8, AKT2, ALMS1, ARMC5, CACNA1D, DIS3L2, EIF2AK3, FOXA2, GATA6, GCG, GCGR, GCK, GLIS3, GLUD1, GPC3, HADH, HNF1A, HNF1B, HNF4A, IGF1, IGF1R, INS, INSR, KCNJ11, KDM6A, LIPE, MC3R, MC4R, NEUROD1, NSD1, PAX4, PDX1, PGM1, PIK3CA, PPARG, PTF1A, RFX6, SH2B1, SIM1, SLC16A1, TUB, UCP2, WFS1, ZFP57

Рахитоподобные заболевания: ADAMTSL2, ALPL, ATP6V0A4, ATP6V1B1, CASR, CLCN5, CLCNKB, CYP24A1, CYP27B1, CYP2R1, DMP1, ENPP1, FAH, FGF23, GALNT3, KL, LRP5, PHEX, PTH1R, SLC2A2, SLC34A1, SLC34A3, SLC9A3R1, VDR

Гипогонадотропный гипогонадизм: ANOS1, BBS1, BBS10, BBS12, BBS2, BBS4, BBS7, BBS9, CHD7, DNMT3L, DUSP6, FEZF1, FGF17, FGF8, FGFR1, FLRT3, GNRH1, GNRHR, HS6ST1, IL17RD, INSL3, KISS1, KISS1R, LEP, LEPR, LHB, MC4R, MKKS, MKRN3, MKS1, MTTP, NR0B1, NSMF, NTRK2, PCSK1, PNPLA6, POLR3A, POLR3B, PROK2, PROKR2, PROP1, RBM28, RNF216, RXFP2, SEMA3A, SH2B1, SIM1, SOX10, SPRY4, TAC3, TACR3, TTC8, WDR11

Аденомы гипофиза: AIP, APC, BRCA1, BRCA2, CDKN1B, CDKN2A, CDKN2C, CHEK2, DICER1, GNAS, MEN1, NF1, POU1F1, PRKAR1A, PRKCA, PTEN, PTTG2, SDHA, SDHB, SDHC, SDHD, TP53

Врожденный гипотиреоз: CACNA1C, DUOX1, DUOX2, DUOXA2, FOXE1, GLIS3, GNAS, IGSF1, IYD, KMT2D, NKX2-1, NKX2-5, PAX8, SECISBP2, SLC16A2, SLC26A4, SLC5A5, TBX1, TG, THRA, THRB, TPO, TRH, TRHR, TSHB, TSHR, TTR, UBR1

Гиперпаратиреоз: AIP, AP2S1, CASR, CDC73, CDKN1A, CDKN1B, CDKN1C, CDKN2A, CDKN2C, CDKN2D, DICER1, FAM111A, GATA3, GCM2, GNA11, GNAS, MEN1, POU1F1, PRKAR1A, PRKCA, PTEN, PTTG2, SDHA, SDHB, SDHC, SDHD, TBCE

Несовершенный остеогенез: BMP1, COL1A1, COL1A2, CRTAP, FKBP10, IFITM5, NFIX, P3H1, PPIB, SERPINF1, SERPINH1, SP7, TMEM38B, WNT1

Примордиальный нанизм: ATR, CCDC8, CDC6, CDT1, CENPJ, CEP152, CEP63, CUL7, DNA2, FBN1, GMNN, NIN, OBSL1, ORC1, ORC4, ORC6, PCNT, PNPLA6, RBBP8, RNU4ATAC, STAT5B, TRAIP

Наследственные липодистрофии: AGPAT2, AKT2, ALB, BSCL2, CAV1, CIDEC, LIPE, LMNA, LMNB2, PIK3CA, PLIN1, POLD1, PPARG, PPP1R3A, PSMB8, TBC1D4, WRN, ZMPSTE24

Нарушения формирования пола: AMH, AMHR2, AR, BMP15, CBX2, CILK1, CYB5A, CYP11A1, CYP17A1, DHCR7, DHH, DMRT1, EMX2, ESR1, ESR2, FEZF1, FGD1, FGF9, FGFR2, FKBP4, FOXF2, FOXL2, FSHR, HOXA13, HSD17B3, HSD3B2, LEP, LEPR, LHCGR, LHX1, LHX9, MAMLD1, MAP3K1, MC4R, MCM9, MID1, MKKS, MKS1, MRPS22, MTOR, MTTP, NR0B1, NR5A1, NTRK2, NUP107, PCSK1, PNPLA6, POR, PROK2, PROKR2, PSMC3IP, PTEN, PTGDS, RSPO1, SH2B1, SOHLH1, SOX9, SRD5A2, SRY, STAR, SUPT3H, TBCE, TSPYL1, WNT4, WT1, ZFPM2

Надпочечниковая недостаточность: AAAS, ABCD1, AIRE, BSND, CACNA1H, CDKN1C, CLCNKA, CLCNKB, CUL3, CYP11A1, CYP11B1, CYP11B2, CYP17A1, DHCR7, H6PD, HSD11B1, HSD11B2, HSD3B2, KCNJ1, LIPA, MC1R, MC2R, MCM4, MRAP, MTTP, NFKB2, NNT, NR0B1, NR3C1, NR3C2, NR5A1, NTRK2, PAPSS2, POMC, POR, SCNN1A, SCNN1B, SCNN1G, SH2B1, SLC26A3, STAR, TBX19, WNK4

Гипогликемия: ACADM, AGL, ALDOB, ALG3, CACNA1C, CACNA1D, CPT1A, CPT2, ETFA, ETFB, ETFDH, FBP1, GHR, HMGCL, KDM6A, MPI, MTOR, PGM1, PMM2, SLC25A32, SLC52A1, SLC52A2, SLC52A3, TBX19

Гипопитуитаризм: ACAN, ARNT2, DLK1, DRD2, FOXA2, GH1, GHRH, GHRHR, GHSR, GLI2, HESX1, IGSF1, LHX3, LHX4, OTX2, PAX6, POU1F1, PROP1, RNPC3, SHH, SOX2, SOX3, TBX19

Феохромоцитома: APC, BRCA1, BRCA2, CHEK2, EGLN1, EGLN2, EPAS1, FH, H3-3A, HRAS, KIF1B, MAX, MDH2, MERTK, MET, NF1, PTEN, RET, SDHA, SDHAF2, SDHB, SDHC, SDHD, TMEM127, TP53, VHL

## Исследование предрасположенности к развитию сахарного диабета 1 типа на основании гаплотипов, а позднее генотипов HLA-системы

В совместных исследованиях, выполненных коллективами сотрудников ЭНЦ, Научно-исследовательского института «Генетика» и Института иммунологии ФМБА России, были получены предварительные данные об этнических различиях ассоциации полиморфных аллелей HLA генов у разных народов России: русских, удмуртов, татар, мари, тувинцев, калмыков, бурятов, якутов. Они выражаются как в специфичности отдельных предрасполагающих и протекторных гаплотипов, так и в их характеристике по показателю относительного риска.

При проведении HLA-типирования в двух русских популяциях (московской и вологодской) в последней было обнаружено повышение частоты встречаемости основного предрасполагающего гаплотипа DRВ1*04-DQA1*0301-DQB1*0302 (11,6% против 8,3%) и достоверное снижение частоты встречаемости протекторного гаплотипа DRВ1*11-DQA1*0501-DQB1*0301 (9,1% против 14,3%). Таким образом, вологодскую популяцию характеризует большая степень генетической предрасположенности и меньший уровень защиты по сравнению с московской популяцией. Эти данные совпадают с результатами популяционных исследований, в которых на основании изучения распределения гаплотипов HLA Архангельская и Вологодская области были объединены в единый кластер с Финляндией, а русские из центральных регионов России — с популяциями из центральной части Европы (при схожем уровне заболеваемости сахарным диабетом 1 типа (СД1) в центральной России и центральной Европе). Как правило, в большинстве этнических групп заболеваемость СД1 значимо ниже, чем среди русского населения, в том числе ниже, чем у русских, проживающих на той же территории. Частота протекторных гаплотипов в финской популяции составляет 1,4–4,1%, у русских из европейской части России — 7,4–14%. Полученные данные демонстрируют большую роль генетических факторов в формировании относительно невысокого уровня заболеваемости СД1 в данной этнической группе. Заметим, что удмурты, по результатам изучения распределения HLA-гаплотипов, были отнесены в кластер Центральной Европы с большой южно-европеоидной примесью [1].

С 70-х гг. XX века стало известно, что СД1 — это аутоиммунное заболевание, возникающее вследствие выработки антител (ICA, IAA, GAD, IA-2, ZnT8) к продуктам жизнедеятельности β-клеток. На основании проведенных исследований HLA и антител была разработана схема индивидуального риска развития СД1, результаты представлены в таблице 2 [2].

**Table:** Таблица 2. Индивидуальный риск развития СД1

Группы	Риск развития СД1
Популяционный риск	0,2%
Братья, сестры больных СД1	6,5%
HLA- идентичные сибсы больных СД1	16–18%
Лица, имеющие предрасполагающие HLA- гаплотипы	18–25%
Лица, имеющие 2 и более антитела (ICA, GADA, IAA, IA2 ZnT8) и предрасполагающие HLA -гаплотипы	90%

Результаты длительного проспективного наблюдения 206 семей, имеющих ребенка с СД1, проведенного Е.В. Титович [3], доказали, что доля сибсов с генотипами высокого риска (DR3/4-DQ2/DQ8) составила 12%, среднего риска (DR/4-DQ8/X или DR3-DQ2/X) — 53%, низкого риска (неDR/4-DQ8/X или неDR3-DQ2/X — 28%), защитными (Х/Х) — 7% (рис. 3). При этом в популяции генотипы высокого риска встречаются у 1,4% людей, низкого риска — у 43%. В результате наблюдения сибсов манифестация заболевания произошла среди детей с генотипами высокого риска в 30% случаев, среднего риска — в 10%, низкого риска — в 3% случаев. Таким образом, из группы высокого риска заболевал каждый 3-й, группы среднего риска — каждый 10-й, группы низкого риска — каждый 30-й сибс. В группе сибсов с защитными генотипами в течение всего 16-летнего периода наблюдения СД1 не развился ни у кого.

**Figure:**
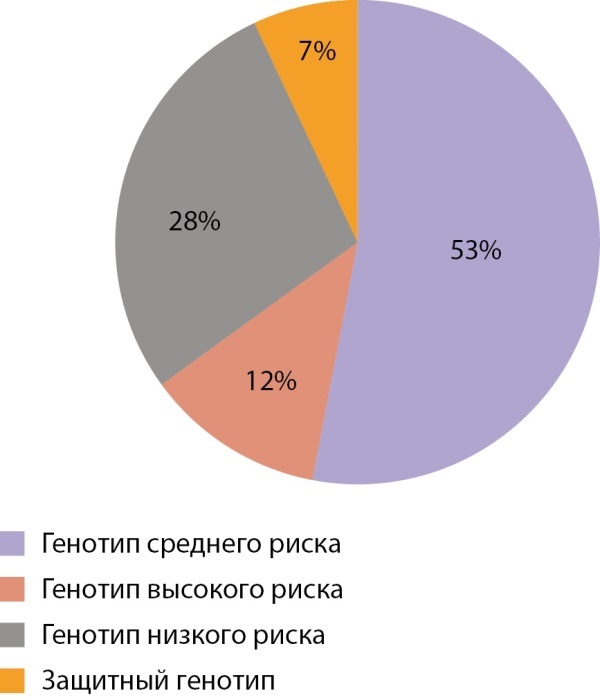
Рисунок 3. Распределение сибсов в семьях с детьми с СД1 по группам генетического риска.

Постулатом XX века был тезис, что сахарный диабет у детей — всегда аутоиммунный, всегда инсулинозависимый и продолжается всю жизнь. Так оно и есть в 90% случаев. Однако в 10% имеет место не аутоиммунное поражение, без инсулинозависимости.

В настоящее время известно более 40 генов, мутации в которых являются причиной неиммунного СД. Наиболее распространенной формой является MODY. Первый отечественный обзор, где содержится упоминание о MODY диабете был сделан О.В. Ремизовым [4], в настоящее время известно уже 14 типов MODY диабета, большинство из которых диагностированы в нашей клинике [5][6][7]. Помимо этого, среди неиммунных форм диабета встречаются более редкие формы: неонатальный сахарный диабет, первое описание в нашей стране принадлежит О.В. Ремизову и соавт. [8], DIDMOAD- синдром и другие. Неонатальный диабет может не требовать инсулинотерапии и быть компенсирован таблетированными препаратами сульфонилмочевины [9]. На рисунке 4 представлена структура моногенных форм СД у детей (n=906), обследованных в Институте детской эндокринологии; среди всех моногенных форм превалируют различные варианты MODY диабета (88%), неонатальный сахарный диабет диагностирован в 6% случаев.

**Figure:**
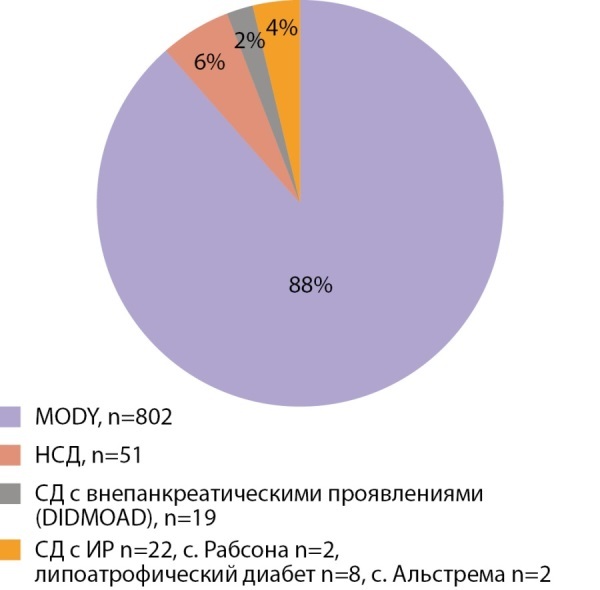
Рисунок 4. Структура моногенных форм СД у детей.

## Второе из трех направлений наших генетических исследований — это молекулярная основа гипофизарной карликовости

Эти исследования начаты О.Ф. Фофановой, А.Н. Тюльпаковым, О.Б. Безлепкиной, Е.В. Нагаевой, О.А. Чикулаевой и продолжены М.С. Панкратовой и другими. Публикации О.В. Фофановой о генах PROP и Pit-1 как причине СТГ-дефицита были первыми в мире [10]. В настоящее время благодаря внедрению в клиническую практику панельнего, экзомного и геномного секвенирования значительно расширились наши знания в этой области. На рисунке 5 представлены частота генетической основы СТГ-дефицита у детей, проанализированных М.С. Панкратовой и соавт. [11].

**Figure:**
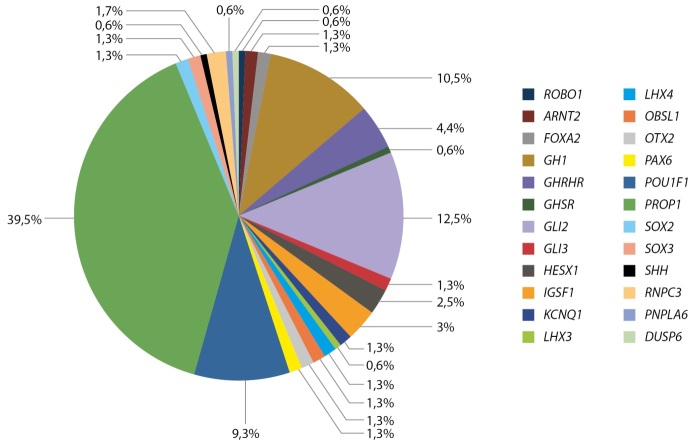
Рисунок 5. Частота различных форм моногенного гипопитуитаризма.

Результаты молекулярно-генетического исследования 657 детей выявили генетическую причину гипопитуитаризма у 24,3% (n=160 детей). Чаще всего встречались варианты в гене PROP1 (n=63, из них 16 сибсов), GH1 (n=17), POU1F1 (n=15), GLI2 (n= 20), GHRHR (n=7). Благодаря проведению полноэкзомного секвенирования, только за последние 5 лет были выявлены 52 пациента с редкими синдромальными формами низкорослости (синдромы Саньяд-Сакати, Рубинштейна-Тейби, Робинова, Ройфмана, Аарского-Скотта, Плавающей гавани, Секкеля и другие).

## Третье направление молекулярно-генетических исследований — диагностика ВДКН

Исследование гена CYP21A2 было начато в 1995 г. С.А. Прокофьевым, Т.В. Семичевой и продолжено М.А. Каревой, А.Н. Тюльпаковым, Н.Ю. Калинченко, И.С. Чугуновым и другими. За последние 10 лет в Институте детской эндокринологии находились под наблюдением более 800 детей с данной патологией из 83 регионов Российской Федерации. Выявлена высокая корреляционная зависимость между генотипом и клиническими проявлениями заболевания, наблюдающаяся у 90% пациентов. Повреждение структуры белка (фермента 21-гидроксилазы) определяет степень его ферментативной активности. Молекулярно-генетическое исследование позволяет не только подтвердить или опровергнуть диагноз, но и дифференцировать форму и с высокой вероятностью (90–95%) предсказать тяжелую сольтеряющую форму заболевания и выбрать схему заместительной терапии. В 2019 г. нами был разработан алгоритм диагностики ВДКН в неонатальном периоде с применением молекулярно-генетического анализа как второго этапа неонатального скрининга, на основании которого, в зависимости от генотипа, выбрана тактика ведения детей с данным заболеванием в неонатальном периоде [12].

## ОРФАННЫЕ ЭНДОКРИННЫЕ ЗАБОЛЕВАНИЯ

Особое место в развитии молекулярной диагностики занимает аутоиммунный полигландулярный синдром 1 типа (АПС1). До начала наших исследований в отечественной литературе было только одно описание этого заболевания [13]. Начиная с 2002 г. Е.М. Орлова и ее ученица Л.С. Созаева диагностировали и наблюдают 205 детей и подростков с АПС1. Это самая большая в мире когорта детей с этим заболеванием, наблюдаемая в одном центре. Ими показано, что самая частая мутация в гене AIRE в Российской Федерации — это р.R257Х, при этом выявлены еще более 20 других мутаций, 10 из которых были описаны впервые в мире, генно-фенотипических корреляций выявлено не было. Л.С. Созаева внедрила в диагностику определение нейтрализующих антител к интерферону-омега, которые показали высокую специфичность (99%) и чувствительность (100%) и антитела к интерферону альфа-2 (специфичность 93,4%, чувствительность 100%). Эти исследования значительно облегчили диагностику этого редкого заболевания и применение современного лечения при нем значительно снизило смертность в детском возрасте [14][15].

Первичная надпочечниковая недостаточность — редкое заболевание, которое у взрослых чаще всего имеет аутоиммунную природу, а в детском возрасте — наследственные причины. Учитывая распространенность наследственных форм у детей, всем детям с установленной первичной надпочечниковой недостаточностью показано проведение генетических тестов. Нами проанализированы данные 88 пациентов с установленной надпочечниковой недостаточностью — 22 девочек и 66 мальчиков. Генетические варианты, объясняющие причину заболевания, установлены у 49 из 88 детей (9 девочек и 40 мальчиков). Самым частым заболеванием, ассоциированным с первичной надпочечниковой недостаточностью, является АПС1 (15 из 49 пациентов, мутации в гене AIRE), вторым по частоте является врожденная гипоплазия надпочечников (14 пациентов, мутации в гене NR0B1), третьим — Х-сцепленная адренолейкодистрофия (11 пациентов, мутации в гене ABCD1). Изолированный дефицит глюкокортикоидных гормонов занимает четвертую позицию по частоте и составляет 14,3% (7 пациентов, генетические варианты гена MC2R выявлены у 6 человек, гена MRAP у 1 человека). Редкими находками был 1 пациент с синдромом Оллгроува (ген AAAS) и 1 пациент с мутацией в гене WT1 с надпочечниковой недостаточностью и гипоспадией (ген WT1) (рис. 6 и 7).

**Figure:**
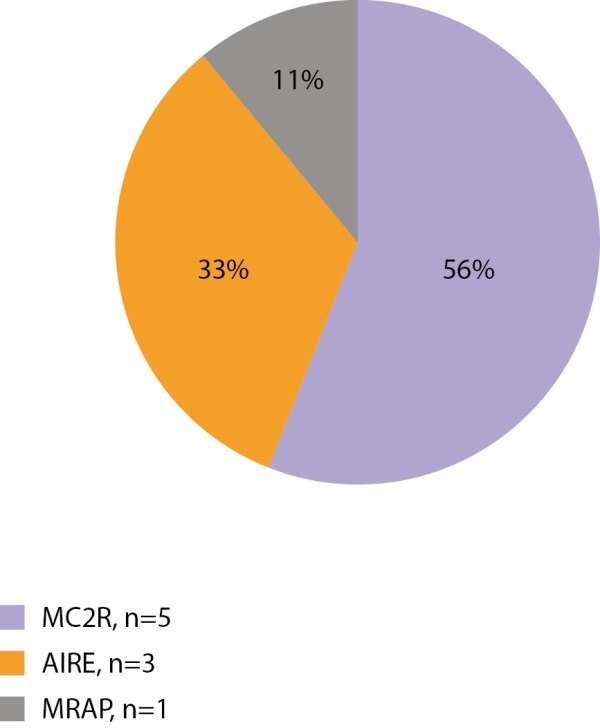
Рисунок 6. Генетическая структура первичной надпочечниковой недостаточности у девочек.

**Figure:**
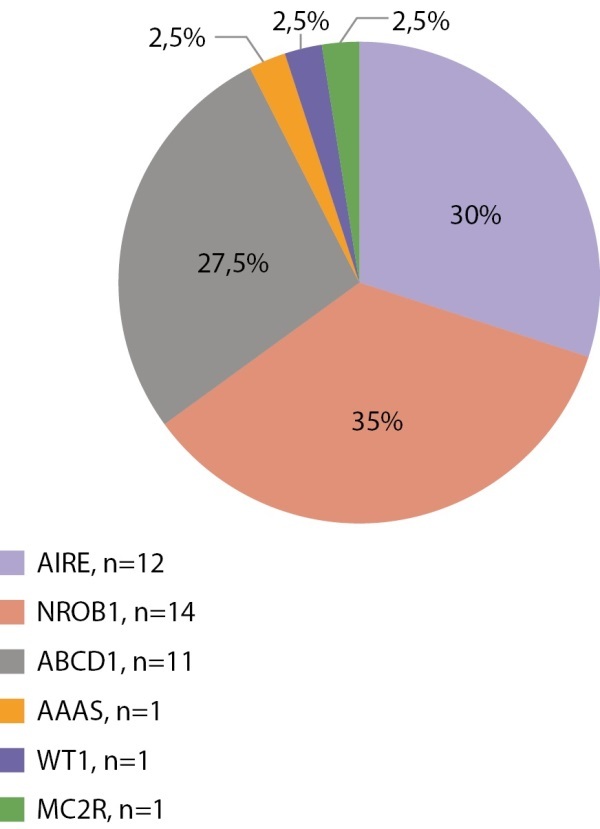
Рисунок 7. Генетическая структура первичной надпочечниковой недостаточности у мальчиков.

Многолетние исследования позволили предложить в клиническую практику алгоритм генетического тестирования при надпочечниковой недостаточности у детей. Мальчикам в первую очередь исключается Х-сцепленная адренолейкодистрофия путем определения уровня очень длинноцепочечных жирных кислот (МГНЦ им. акад. Н.П. Бочкова) или проведения секвенирования гена ABCD1 методом Сэнгера в нашем центре. Если у пациента имеют место типичные для определенного наследственного заболевания компоненты, то проводится прицельное исследование предполагаемого гена методом Сэнгера [16][17]. Например, если имеется сочетание первичной надпочечниковой недостаточности и гипопаратиреоза, то методом выбора будет секвенирование гена AIRE. В большинстве случаев образцы крови пациентов направляются на исследование с применением панели «Надпочечниковая недостаточность».

Гипогонадотропный гипогонадизм — это группа заболеваний, обусловленных нарушением продукции, дискретной секреции или действия гонадотропин-рилизинг-гормона. Большинство случаев гипогонадотропного гипогонадизма встречается у пациентов мужского пола. Клинически гипогонадотропный гипогонадизм проявляется задержкой пубертата и снижением фертильности. Выделяют врожденную и приобретенную форму гипогонадотропного гипогонадизма. В настоящее время известно более 60 генов, вариантные замены в которых приводят к развитию врожденного гипогонадотропного гипогонадизма [18]. Данную группу заболеваний характеризует высокая вариабельность фенотипа, которая может проявляться в рамках одной семьи, что делает трудным прогнозирование течения гипогонадизма в подростковом и взрослом возрасте.

В настоящее время проводится изучение этиологии гипогонадизма с применением панели «Гипогонадотропный гипогонадизм», содержащей 53 гена. На рисунке 8 представлены результаты процентного соотношения выявленных генов, обследованных по данной панели. Использование панели в 58% случаев позволило выявить генетическую природу заболевания. Наиболее часто встречались патогенные и вероятно патогенные варианты в генах ANOS1 (19%), GNRHR (16%) и FGFR1 (13%).

**Figure:**
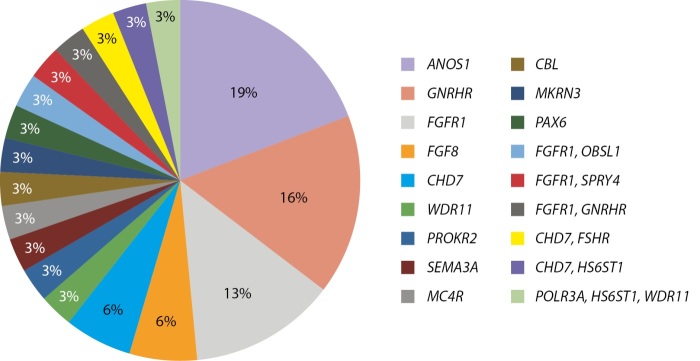
Рисунок 8. Молекулярно-генетические причины гипогонадотропного гипогонадизма у мальчиков.

Врожденный гиперинсулинизм — редкое, сложное этиологически и генетически гетерогенное заболевание. К сожалению, поздняя диагностика и неадекватная терапия приводят к инвалидизации пациентов. Частота заболеваний в Российской Федерации в настоящее время, благодаря работам нашего института, начатым М.А. Меликян, составляет 1:50 000, что совпадает с данными исследований в странах Европы и США. Молекулярно-генетические исследования были проведены у 214 пациентов, при этом молекулярно-генетическую причину заболевания удалось установить у 146 пациентов (68,2%), результаты представлены на рисунке 9.

**Figure:**
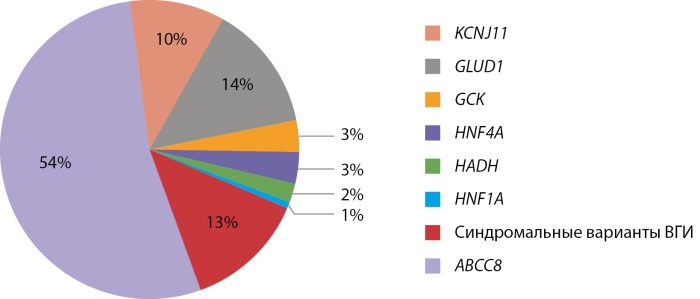
Рисунок 9. Данные молекулярно-генетического исследования пациентов с ВГИ.

Всего в генах калиевых каналов выявлено 93 патогенных варианта, 33 из которых ранее в мировой литературе не описаны. Выявлена выраженная гетерогенность механизмов наследования мутаций, определяющих морфологический вариант и тактику ведения [19].

В результате многолетних исследований был разработан алгоритм диагностики и персонализированного лечения врожденного гиперинсулинизма, в котором ведущее место отводится молекулярно-генетическому исследованию, пробному лечению диазоксидом. Определены показания к проведению ПЭТ/КТ с 18FДОФА, что позволяет выявить диффузные или фокальные формы и определить тактику хирургического лечения: при фокальной форме — частичная резекция, что приводит к полному выздоровлению, или субтотальная резекция с развитием сахарного диабета.

Эндокринные новообразования в детском и подростковом возрасте образуют гетерогенную группу орфанных заболеваний. При обнаружении определенных видов опухолей у детей, таких как опухоли надпочечников, гормон-секретирующие аденомы гипофиза, медуллярный рак щитовидной железы и другие, требуется проведение молекулярно-генетических исследований для исключения моногенных, семейных вариантов опухолевых синдромов. В рамках программы «Альфа-Эндо» проводится диагностика большинства наследственных синдромов, для которых характерно наличие опухолей эндокринных желез. При ряде заболеваний, например, при синдроме множественных эндокринных неоплазий 2 типа, выявление патогенных вариантных замен может определять не только протокол динамического наблюдения, но и выбор активной хирургической тактики. Так, в 2001 г. В.Г. Поляковым проведена первая в России превентивная тиреоидэктомия у двух здоровых детей из нашей клиники при доказанной мутации в гене RET — протоонкогена. В настоящее время превентивная тиреоидэктомия в этих случаях — золотой стандарт [20].

С 2014 г. в Институте детской эндокринологии проводится изучение молекулярно-генетической этиологии врожденного гипотиреоза с использованием панели генов «Врожденный гипотиреоз», включающей 28 генов. С целью углубленного изучения молекулярно-генетических нарушений при врожденном гипотиреозе было проведено полноэкзомное секвенирование образцов цельной крови пациентов (116 детей с дисгенезией щитовидной железы и 12 с дисгормогенезом). На рисунке 10 представлены полученные данные в зависимости от топографии ЩЖ, определенной по результатам сцинтиграфии.

**Figure:**
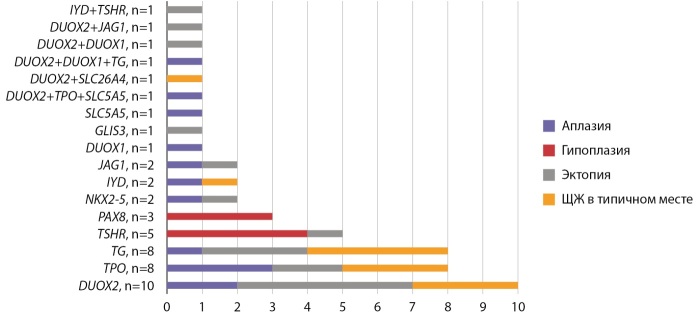
Рисунок 10. Изменения в генах, связанные с врожденным гипотиреозом в зависимости от топографии ЩЖ.

Патогенные и вероятно патогенные варианты были выявлены у 38,3% детей, в 61,2% случаях в генах, ассоциированных с биосинтезом тиреоидных гормонов (DUOX2, TPO, TG), в 26,6% — в генах, участвующих в закладке и миграции ЩЖ (TSHR, PAX8, JAG1, NKX2-5, GLIS3), в 12,2% — различные дигенные и олигогенные варианты. Интересно, что гены «дисгормоногенеза» были найдены и при различных вариантах дисгенезии ЩЖ, что может свидетельствовать об их участии в закладке и миграции тиреоидной ткани, помимо экспрессии соответствующих ферментов гормоногенеза [21][22][23]. Полное соответствие «генотип-тиреоидный фенотип» было выявлено только у пациентов с гипоплазией ЩЖ.

Актуальной проблемой является преимплантационная генетическая диагностика, цель которой — в семье, имеющей доказанную генетическую мутацию при определенном заболевании, отобрать эмбрион без генетических нарушений. Этот метод в будущем позволит значительно снизить частоту рождения детей с тяжелыми генетическими заболеваниями. Экономическая выгода этого метода очевидна и трудно поддается подсчету. Например, лечение ребенка с мукополисахаридозом обходится государству в 20 000 000 рублей в год. Кроме того, в данном роду будет прервана генетическая передача данного заболевания. Необходима большая просветительская работа среди врачей и в семьях, где есть ребенок с генетически тяжелым заболеванием.

Подытоживая сказанное, можно констатировать, что молекулярно-генетические исследования в отечественной детской эндокринологии стали почти рутинным методом, доступным всем врачам и детям с эндокринопатиями. Заглядывая в будущее, верим, что недалеко то время, когда станет возможным редактировать (исправлять) мутированные гены и вылечивать многие моногенные эндокринные заболевания.

## ДОПОЛНИТЕЛЬНАЯ ИНФОРМАЦИЯ

Конфликт интересов. Авторы декларируют отсутствие явных и потенциальных конфликтов интересов, связанных с содержанием настоящей статьи.

Участие авторов. Петеркова В.А., Безлепкина О.Б. — аналитическая работа и подготовка финальной версии статьи, Панкратова М.С., Чугунов И.С., Лаптев Д.Н., Нагаева Е.В., Ширяева Т.Ю., Колодкина А.А., Созаева Л.С., Титович Е.В., Болмасова А.В., Кураева Т.Л. — редактирование текста, внесение ценных замечаний. Все авторы одобрили финальную версию статьи перед публикацией, выразили согласие нести ответственность за все аспекты работы, подразумевающую надлежащее изучение и решение вопросов, связанных с точностью или добросовестностью любой части работы.
